# Precision Phenotyping With Audiometric Data and Gene Discovery for Sensorineural Hearing Loss

**DOI:** 10.1001/jamaoto.2026.3089

**Published:** 2026-10-08

**Authors:** Andrea J. DeFreese, Tanguy Rubat du Mérac, Quanhu Sheng, Srishti Nayak, Taha A. Jan

**Affiliations:** 1 Department of Hearing and Speech Sciences, Vanderbilt University, Nashville, Tennessee; 2 Department of Otolaryngology–Head and Neck Surgery, Vanderbilt University Medical Center, Nashville, Tennessee; 3 Human Genetics Program, Vanderbilt School of Medicine, Vanderbilt University, Nashville, Tennessee; 4 Vanderbilt Genetics Institute, Vanderbilt University Medical Center, Nashville, Tennessee; 5 Department of Biostatistics, Vanderbilt University Medical Center, Nashville, Tennessee; 6 Center for Quantitative Sciences, Vanderbilt University Medical Center, Nashville, Tennessee; 7 Department of Cell and Developmental Biology, Epithelial Biology Center, Vanderbilt Center for Stem Cell Biology, Center for Computational Systems Biology, Vanderbilt University Medical Center, Nashville, Tennessee

## Abstract

**Importance:**

The genetic architecture underlying hearing sensitivity as a quantitative trait is critical for advancing precision medicine in hearing health. Despite extensive genome-wide association study (GWAS) efforts, few strong genetic drivers have been identified.

**Objective:**

To compare the sensitivity for detecting genetic variants associated with sensorineural hearing loss (SNHL) using precision phenotyping with pure-tone averages (PTAs) with common phenotyping using diagnostic coding approaches.

**Design, Setting, and Participants:**

In this genetic association study, 2 separate GWASs were conducted using different phenotyping approaches for SNHL: (1) diagnostic code control and (2) precision phenotyping with PTA data. Whole-genome sequencing data from a population with European genetic ancestry collected at a single tertiary center biobank was used. For the control GWAS based on diagnostic codes, case and control participants were defined using primarily *International Classification of Diseases, Ninth Revision *(*ICD-9*) and* International Statistical Classification of Diseases and Related Health Problems, Tenth Revision *(*ICD-10*) diagnosis and procedure codes related to SNHL hearing loss. For the precision phenotyping GWAS, hearing status was defined as a continuous variable, using the standard 3-frequency PTA of the better hearing ear from the audiogram. Data analysis was conducted from August 2025 to April 2026.

**Main Outcomes and Measures:**

GWAS summary statistics were used to calculate estimates of single-nucleotide variant (SNV)–based heritability for PTA and *ICD* phenotypes; to map the genetic risk loci to candidate genes using functional annotation; to evaluate genetic correlations with existing GWASs; and to calculate polygenic risk scores for testing in an independent cohort from the National Institutes of Health’s All of Us Research Program.

**Results:**

Overall, 10 164 case participants (mean [SD] age, 63.9 [21.3] years; 5118 [50.4%] female) and 51 305 control participants (mean [SD] age, 51.1 [21.0] years; 28 784 [56.1%] female) were included in the first GWAS; 16 057 participants with PTA (mean [SD] age, 55.5 [21.8] years; 9147 [57.0%] female) were included in the second GWAS. PTA-based GWAS identified 3 genome-wide significant loci mapped to 4 genes: *EML6*, *SPTBN1*, *ARHGEF28*, and *EYA4* (SNV-based heritability estimate [*h*^2^] = 11.78%; SE = 2.84%). *ICD*-based GWAS identified no significant loci (*h*^2^ = 2.90%; SE = 0.77%). Genome-wide correlations between the PTA-based GWAS and 3 hearing-related ability traits were found (eg, PTA-based GWAS and hearing aid use: *r* = 0.985; SE = 0.163; *P* = 1.56 × 10^−9^). In the All of Us cohort, PTA-derived polygenic risk scores were significantly associated with self-reported deafness (odds ratio [OR], 1.09; 95% CI, 1.07-1.12), whereas *ICD*-derived scores were not (OR, 1.00; 95% CI, 0.97-1.02).

**Conclusions and Relevance:**

In this genetic association study, precision phenotyping using audiometric PTAs improved genetic discovery for SNHL compared with diagnostic code–based approaches. Higher heritability estimates, identification of genome-wide significant loci, and stronger polygenic risk estimation indicate that quantitative hearing measures better capture genetic architecture, supporting precision medicine in hearing health.

## Key Points

**Question:**

Does precision phenotyping for sensorineural hearing loss improve the characterization of its genetic architecture?

**Findings:**

In this genetic association study, precise phenotyping of sensorineural hearing loss using pure-tone averages (PTAs) in 16 057 participants yielded greater genetic discovery than traditional diagnostic code–based phenotyping in 61 469 participants. Specifically, PTA-based GWAS yielded more genetic loci reaching genome-wide significance (3 vs none), more robust heritability estimates (11.87% vs 2.9%), and polygenic risk scores that provided better estimation of hearing-related outcomes.

**Meaning:**

In this study, genetic discovery and polygenic risk estimation for sensorineural hearing loss were enhanced by precision phenotyping with physiologic audiometric data, with the potential to improve individualized risk assessment and inform earlier diagnosis and preventive or therapeutic strategies.

## Teaser

This genetic association study compares the performance of a traditional, diagnosis-based genome-wide association study (GWAS) with a GWAS using precision phenotyping and pure-tone averages for identifying genetic drivers and individuals at risk of sensorineural hearing loss.

## Introduction

Hearing loss is the most common sensory disorder. It affects 1.5 billion individuals globally, a number projected to reach 2.5 billion by 2050.^1^ If left undiagnosed, hearing loss negatively impacts both the individual and the world around them. For individuals, it disrupts communication,^2^ education,^3^ and employment opportunities,^4^ all of which contribute to social isolation and diminished quality of life. Furthermore, if left untreated, hearing loss is associated with an increased risk of dementia.^6^ On a global scale, untreated hearing loss results in nearly $1 trillion in annual costs due to lost productivity and health care expenditures.^1^ These outcomes underscore the urgent need for earlier diagnosis and more effective treatments to prevent these adverse effects.

Genetics offers an opportunity to identify individuals at risk for hearing loss before symptoms manifest, enabling early intervention. Thus far, large-scale genome-wide association study (GWAS) efforts^10^ have identified a range of genetic influences underlying hearing impairment, revealing modest single-nucleotide variant (SNV) heritability.^18^ However, one important limitation remains that many GWAS analyses rely on readily available electronic health record (EHR) data. A meta-analysis of hearing-loss GWAS reported that across 17 independent cohorts, hearing loss was typically defined using self-report or diagnostic codes, phenotypes that are often subjective and imprecise.^20^ In addition, these approaches frequently treat hearing loss as a binary trait, failing to capture the multidimensional nature of hearing sensitivity, which varies across frequency (eg, low vs high), severity (eg, mild to profound), and configuration (eg, sensorineural vs conductive). Therefore, to capture this complexity, these GWAS efforts may benefit from what we are calling precision phenotyping: the use of granular, quantitative, or multidimensional trait measurements rather than broad diagnostic categories to more accurately capture an individual’s disease state.

Several studies have utilized audiometric data to apply precision phenotyping to hearing loss to assist genomic discovery. While these demonstrate the value of objective audiometric data and have identified several suggestive loci associated with hearing sensitivity, these analyses have been conducted in relatively small or moderately sized cohorts, limiting statistical power to detect genome-wide significant associations.^13^ In contrast, larger studies have often simplified hearing sensitivity into binary traits derived from air-conduction thresholds or partitioned cohorts into smaller groups, approaches that may reduce sensitivity for detecting genetic effects, introduce heterogeneity from conductive mechanisms, or reduce effective sample size within each analysis.^12^

Together, these prior studies highlight both the promise and the challenges of audiometric phenotyping for hearing-loss gene discovery. Whether precision phenotyping using quantitative audiometric measures, rather than diagnostic codes, enhances genetic discovery for SNHL remains unknown. Furthermore, the lack of availability of audiometric data for precision hearing phenotypes in large-scale EHRs has impeded progress in this research area. To address this knowledge gap, we asked: does precision phenotyping enhance genetic discovery and downstream polygenic risk estimation compared with diagnostic code–based phenotyping?

The present study addresses this question by comparatively evaluating the following features across diagnostic-based and audiometry-based GWASs in Vanderbilt’s EHR-linked biobank, BioVU: (1) discovery of genome-wide significant loci associated with SNHL and (2) validation of genome-wide genetic signal in an independent large cohort, All of Us (eg, polygenic risk score [PRS] analyses; genetic correlations with existing hearing-related GWASs). We hypothesize that precision phenotyping using pure-tone averages (PTAs) derived from audiometric data will provide greater sensitivity for detecting genetic signal associated with SNHL compared with diagnostic coding approaches and will show more validity (eg, more robust polygenic associations with hearing phenotypes in an independent large cohort). By modeling hearing sensitivity as a quantitative (continuous) trait, this study advances precision medicine research efforts for hearing health.

## Methods

### Ethics

All participants in BioVU, Vanderbilt University Medical Center’s deidentified biobank, provided informed consent during initial enrollment. Use of these data was approved by the Vanderbilt University Medical Center Institutional Review Board (No. 221125). All participants in the All of Us Research Program provided written informed consent, and access to these data for PRS analyses was conducted in accordance with program data use policies.^26^ Data analysis was conducted from August 2025 to April 2026.

### Phenotyping

Two cohorts for SNHL were created: one using diagnostic code–based phenotyping and another using audiometric pure tone–based precision phenotyping. Both cohorts were restricted to individuals of European genetic ancestry, as this was the largest ancestry group in the sample.

#### Diagnostic Code–Based Phenotyping

Case and control definitions followed the PheKB SNHL phenotype definition^27^ (eMethods and eFigure 1 in Supplement 1), which uses a combination of *International Classification of Diseases, Ninth Revision (ICD-9)*, *International Statistical Classification of Diseases and Related Health Problems, Tenth Revision (ICD-10)*, and *Current Procedural Terminology *(*CPT*) codes as well as medical record notes extracted from the Synthetic Derivative, a deidentified version of Vanderbilt University Medical Center’s EHR. In this definition, phenotyping looks for the presence or absence of documented SNHL, regardless of severity, laterality, or configuration. For this cohort, age was defined as the age at time of diagnosis (case participants) or the most recent medical record encounter (control participants). Data were filtered to only include individuals older than 3 years to reflect a postlingual onset of hearing loss.

#### Audiometric-Based Precision Phenotyping

Deidentified audiometric data were obtained from 153 826 patients in Vanderbilt University Medical Center’s clinical database (eFigure 1 in Supplement 1). For each patient, a 3-frequency PTA was calculated using unaided air- and bone-conduction thresholds at 500, 1000, and 2000 Hz for both ears. These frequencies were selected to reflect clinical PTA conventions^28^ and to reduce potential confounding from high-frequency noise-induced hearing loss.^30^ The PTA from the better-hearing ear, defined as the ear with the lower air-conduction PTA, was used for all analyses. For patients with multiple audiograms, the most recent audiogram was selected; for patients who subsequently received a cochlear implant, the most recent audiogram prior to implantation was used.

Patients with purely conductive hearing loss in either ear were excluded. Conductive hearing loss was defined as the presence of an air-bone gap of 15 dB or greater together with a bone-conduction PTA of 25 dB or less at the same frequencies. For this cohort, age was defined as the age at the most recent audiogram used to calculate PTA.

### Genotyping and Quality Control

Analyses were based on the 250 000 whole-genome sequencing release of the Alliance for Genomic Discovery in BioVU.^33^ Details for sequencing, genotyping, and quality control have been previously reported for the flagship cohort.^34^ In brief, whole-genome sequencing was performed on Illumina platforms with alignment to the GRCh38 reference genome. The small variants were called using DRAGEN Germline Variant Calling Pipeline version 3.7.8, recalibrated using DRAGEN Machine Learning (ML) Recalibration pipeline version 4.2.4, and aggregated using DRAGEN Iterative gVCF Genotyper pipeline version 4.3.7. Variants with recalibrated variant quality (QUAL) less than 3.0 are filtered before the aggregation. For variants with genotyping rate less than 90%, the FILTER is set as LowGTR, and QUAL at 0. For variants with genotyping rate greater than 90%, the cohort-level ML filtering is performed based on genotyping rate and genotyping quality from all samples. The cohort level ML filtering calculates an ML site quality (MLSQ) and set as QUAL. For sites with QUAL of 0.1 or greater, the FILTER is set as PASS and otherwise as LowMLSQ. Only the PASS variants were used in this project. Principal components (PCs) of genetic ancestry were derived using reference populations from the 1000 Genomes Project, and analyses in the present study were restricted to individuals of European genetic ancestry.

### Statistical Analysis

#### Genome-Wide Association Analyses

GWAS was conducted using REGENIE,^35^ which leverages a 2-step process to estimate genetic effects of individual variants on the selected hearing phenotypes. In step 1, genomic predictions were derived to account for sample relatedness and population structure using a linkage disequilibrium–pruned set of variants (*r*^2^ of approximately 0.45). These predictions were then incorporated in the second step to model genetic associations across the full set of common variants.

Quality control included removing samples with missingness greater than 10% as well as variants with missing call rate greater than 10%, minor allele count (MAC) less than 100, minor allele frequency (MAF) less than 0.01, or Hardy-Weinberg equilibrium *P* < 1 × 10^−15^. Multiallelic and non-ACGT variants were excluded from all analyses, and analyses included both autosomes (chromosomes 1-22) and chromosome X. In step 1, we used a genotype block size of 1000 variants, followed by association testing in step 2 using a block size of 400 variants and a leave-one-chromosome-out approach. For binary traits, we applied a Firth likelihood ratio test correction for variants with *P* < .01 to account for case-control imbalances.

Separate GWAS were conducted for *ICD*-based binary phenotypes and PTA-based continuous traits. PTA measures were inverse rank-normalized prior to analysis to address violations of the normality assumption (eFigure 2 in Supplement 1). All models were adjusted for age at diagnosis (*ICD*-based) or audiologic testing (PTA-based), biological sex, and the first 10 ancestry-informative genetic PCs and were restricted to individuals of European genetic ancestry. These steps yielded distinct GWAS summary statistics for the *ICD*-based and PTA-based phenotypes.

#### Functional Annotation of Top Associated Loci

We used functional mapping and annotation (FUMA)^36^ to perform functional variant annotation of the top associated loci of our GWAS results. Genome-wide significance was defined using the standard threshold of *P* < 5 × 10^−8^. Suggestive significance was defined at *P* < 1 × 10^−5^.

#### Linkage Disequilibrium Score Regression Analysis

Using linkage disequilibrium score regression (LDSC),^37^ we estimated genetic correlations between our GWAS summary statistics and the following hearing-related traits with published GWAS summary statistics: hearing aid use,^15^ PTA,^13^ *ICD* and self-report of hearing loss,^21^ and speech recognition in noise.^15^ Significance after adjustment for multiple comparison was established using Bonferroni-correction at *P* < .0125 (.05/4 tests).

#### Validation via Polygenic Risk Scores in All of Us

To validate the GWAS signal, we derived PRS for SNHL and evaluated whether the risk scores were associated with self-reported hearing loss in the National Institutes of Health’s All of Us. For comparison, the main factors capturing the polygenic signal for SNHL were standardized and built on the 2 GWAS summary statistics derived from the PTA precision phenotyping and the *ICD*-based phenotyping. All PRS models were adjusted for age at last *ICD* code assignment, sex at birth, and the first 5 ancestry-informative genetic PCs that captured the majority of ancestry-related variation.

##### All of Us Sample (Curated Data Repository Version 8)

The All of Us Research Program previously enrolled adult participants (age ≥18 years) at multiple clinics and regional medical centers distributed across the United States. A more detailed overview of the All of Us initiative can be found elsewhere.^26^ We registered this project within the All of Us Researcher Workbench, accessing the C2024Q3R4 Curated Data Repository version 8 (Controlled Tier), which includes participant data obtained up to October 1, 2023. Data accessed consisted of individual-level EHR data, including medical diagnostic codes (eg, *ICD-9* and *ICD-10*), procedure codes (eg, *CPT*), short-read whole-genome sequencing (srWGS), and health survey data and demographic information, such as age and sex.

##### Definition of Case and Control Participants in All of Us

Hearing loss case participants, defined as having self-reported hearing loss, were identified with the survey response to 1 hearing loss question in *All of Us* (N = 109 096): (1) “Are you deaf or do you have serious difficulty hearing?” A total of 9365 case participants selected yes, and 99 731 control participants selected no. We selected this item because participants were able to explicitly report presence or absence of hearing loss.

##### Quality Control

Extensive quality control was applied to the srWGS data made available through the All of Us Research Program^26^ using PLINK version 1.9 ^39^ or 2.0.^40^ All analyses were restricted to individuals of European genetic ancestry. Monomorphic, duplicated, multiallelic, and non-ACGT variants were excluded, along with outlier samples previously flagged within the public release. Samples with missingness greater than 5% and variants with missing call rate greater than 5%, MAF less than 0.01, or Hardy-Weinberg exact test of *P* < 1 × 10^−6^ were removed, resulting in a high-quality srWGS dataset merged across autosomes.

##### PC Analysis

We conducted LD pruning on the final quality-controlled srWGS data resulting in a total set of 120 789 markers. Ancestry-informative genetic PCs were derived using PLINK2,^40^ adjusting for participant kinship. We used KING^41^ to infer participant’s relatedness (kinship coefficient, 0.0442) which resulted in 16 707 of 234 069 samples (7.14%) samples flagged as related up to the third degree. Relatedness was accounted for within the PC generation, but related samples were not excluded from the PRS modeling.

## Results

### Demographic Characteristics

A total of 10 164 case participants (mean [SD] age, 63.9 [21.3] years; 5118 [50.4%] female) and 51 304 control participants (mean [SD] age, 51.1 [21.0] years; 28 784 [56.1%] female) were included in the *ICD*-based cohort, and 16 057 participants (mean [SD] age, 55.5 [21.8] years; 9147 [57.0%] female), with a range of hearing from normal hearing to profound hearing loss by PTA (eFigure 2 in Supplement 1) were included in the PTA precision phenotyping cohort. Between datasets, 7479 participants were shared. Age and sex are summarized in Table 1.

**Table 1.** Participant Demographic Characteristics by Genome-Wide Association Study Cohort

Characteristic	*ICD* cohort		PTA cohort
	Case participants	Control participants	
Total No.	10 164	51 304	16 057
Age, mean (SD), y	63.89 (21.29)	51.08 (21.00)	55.52 (21.77)
Sex assigned at birth, No. (%)			
Female	5118 (50.4)	28 784 (56.1)	9147 (57.0)
Male	5046 (59.6)	22 520 (43.9)	6910 (43.0)

Abbreviations: *ICD*, *International Classification of Diseases*; PTA, pure-tone average.

### GWAS

Genome-wide association analyses were well calibrated, with no evidence of inflation (*ICD* genomic inflation factor [λ_GC_] = 1.03; PTA λ_GC_ = 1.04). In the *ICD* cohort, no loci reached genome-wide significance (*P* < 5 × 10^−8^) (Figure 1A). In the PTA cohort, 3 loci met genome-wide significance (Figure 1B) and were mapped by functional annotation to 4 genes: *EML6 *(OMIM 621084), *SPTBN1* (OMIM 182790), *ARHGEF28 *(OMIM 612790), and *EYA4* (OMIM 603550) (Table 2). A total of 41 and 21 loci reached the level of suggestive significance (*P* < 1 × 10^−5^) in the PTA-based and *ICD*-based GWASs, respectively.

**Figure 1.**
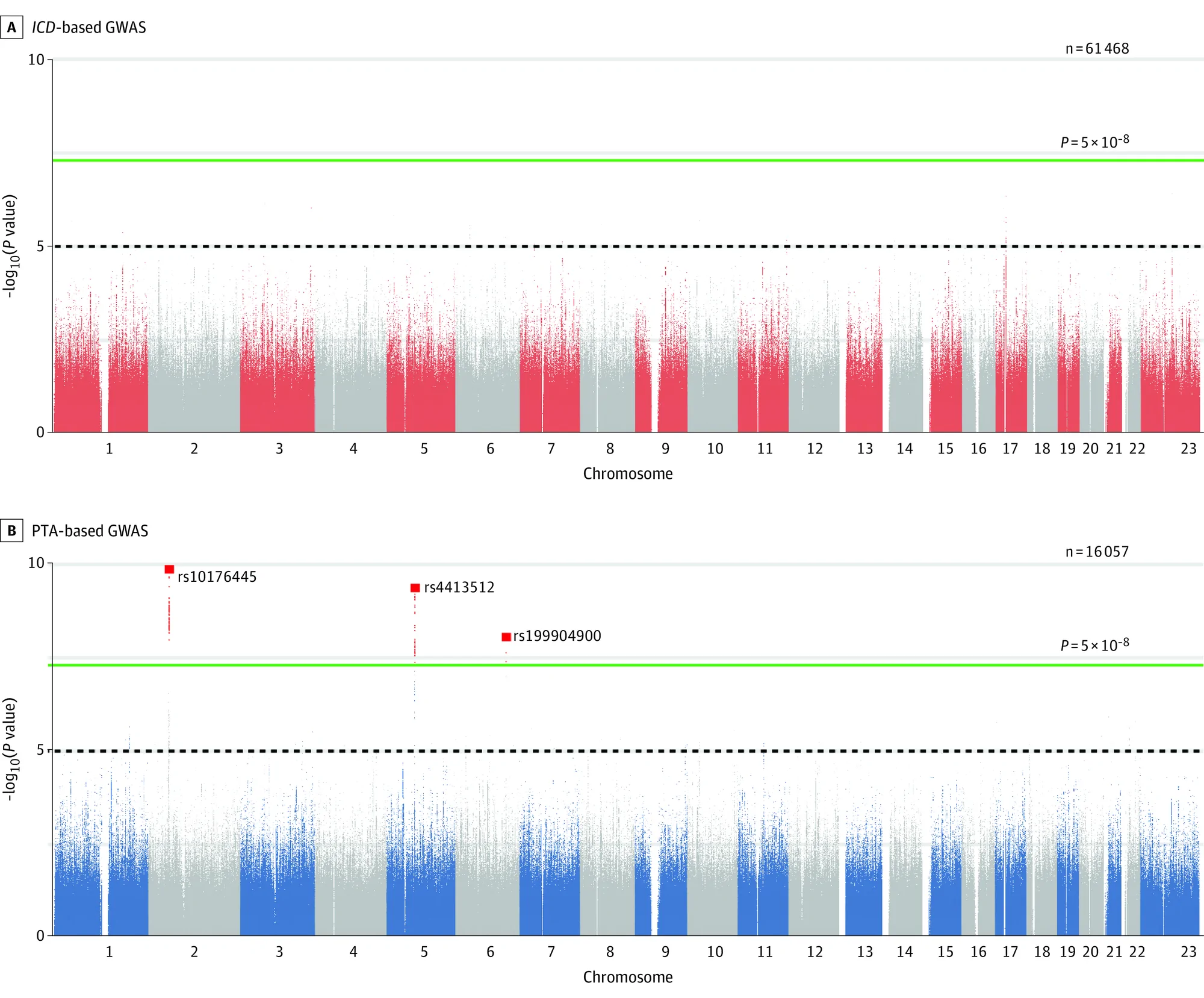
Scatterplot of Genetic Architecture of Sensorineural Hearing Loss Based on *International Classification of Diseases *(*ICD*) and Pure-Tone Average (PTA) Definitions Genome-wide association study (GWAS) plots based on precision phenotyping (PTA) and diagnostic codes (*ICD*) across all autosomes (chromosomes 1-22) and chromosome X. A, The GWAS included 61 468 individuals using an *ICD*-based definition of clinical case vs control participants. The GWAS included 16 057 participants using quantitative PTA measures. For both panels, genome-wide significance is represented by the plain green line (*P* = 5 × 10^−8^) and an alternative (suggestive) significance threshold is depicted by a dashed line at* P* = 1 × 10^−5^.

**Table 2.** Summary Statistics for the Top Single-Nucleotide Variants Associated With Pure-Tone Average

rsID	Chromosome	β (SE)	*P* value	Gene	Biological impact
rs10176445	2	0.071 (0.011)	1.33 × 10^−10^	*EML6*	Involved in microtubule dynamics of nervous system and sensory organs
				*SPTBN1*	Involved in central nervous system development and function
rs4413512	5	0.058 (0.009)	4.21 × 10^−10^	*ARHGEF28*	Involved in axonal branching and synapse formation
rs199904900	6	0.057 (0.009)	8.92 × 10^−9^	*EYA4*	Involved in organ of Corti function; associated with nonsyndromic SNHL

Abbreviations: rsID, single-nucleotide variant identification number; SNHL, sensorineural hearing loss.

To ensure the validity of our comparative approach, we aimed to establish that significant genetic signal explained variability in our 2 primary SNHL phenotyping definitions of PTA and *ICD*-based coding. We found both phenotyping approaches produced significant SNV-based heritability (*h*^2^) estimates, albeit reaching notably different orders of magnitude. LDSC estimated the heritability of the PTA and *ICD*-based GWAS SNHL to reach an *h*^2^of 11.78% (SE, 2.84%) and 2.90% (SE, 0.77%), respectively. We observed a weak but statistically significant correlation between *ICD* codes and PTA in the group of participants who appeared in both cohorts (7479 participants; point-biserial *r* = 0.106).

To further validate the GWAS signal derived from the precision phenotyping, we estimated genome-wide correlations between our PTA-based GWAS of SNHL and 4 distinct hearing-related ability traits (Figure 2). We identified significant genetic overlap between our PTA-based GWAS and clinically diagnosed and self-reported hearing loss^21^ (*r* = 0.570; SE = 0.089; *P* = 1.31 × 10^−10^), hearing aid use^15^ (*r* = 0.985; SE = 0.163; *P* = 1.56 × 10^−9^), and a previously conducted GWAS of PTA based on a smaller sample of 9675 participants^13^ (*r* = 0.9376; SE = 0.3462; *P* = .007). No statistically significant genetic correlation was observed with speech-in-noise perception,^15^ with the estimate being consistent with negligible-to-no genetic overlap between the 2 traits (*r* = 0.046; SE = 0.091; *P* = .61).

**Figure 2.**
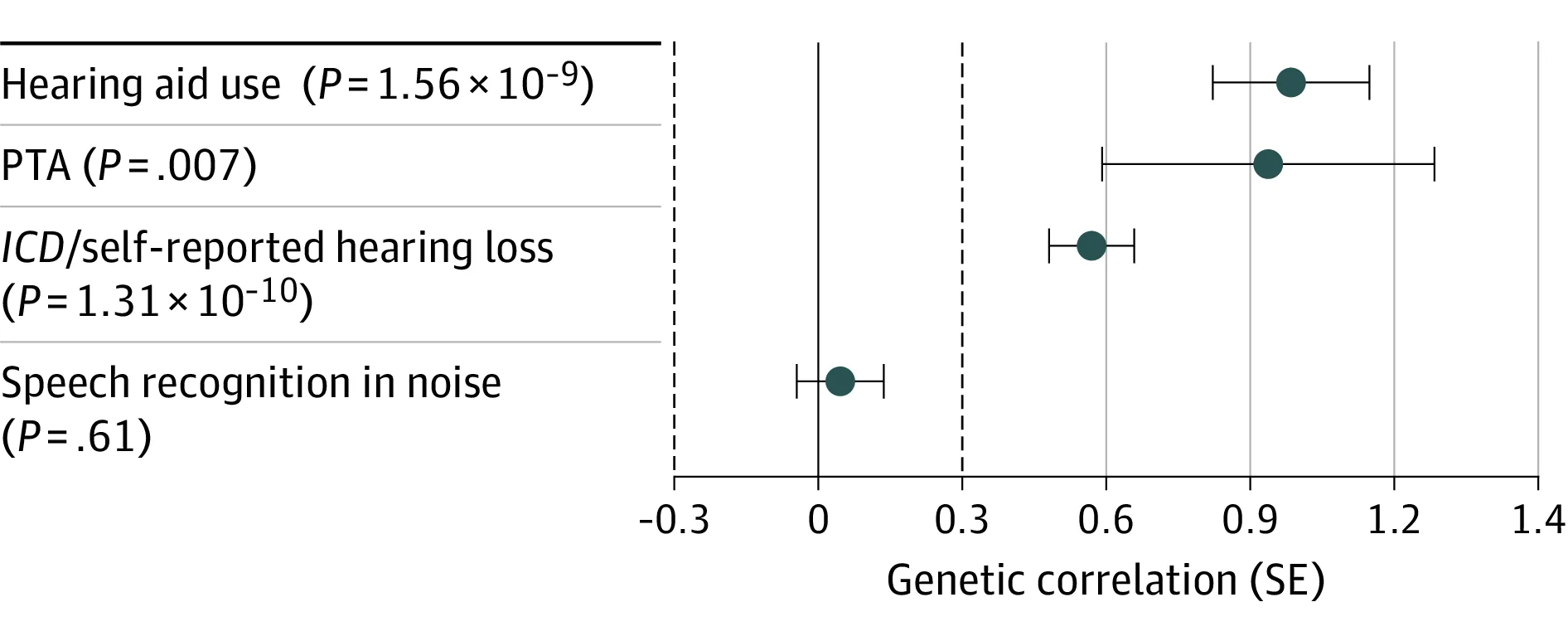
Dot Plot of Genetic Correlations With Other Hearing-Related Traits Dot plot of genetic correlations with SEs, estimated via linkage disequilibrium score regression between a genome-wide association study of postlingual sensorineural hearing loss (pure-tone average–derived) and 4 other genome-wide association studies of hearing related traits: hearing aid use,^15^ pure-tone average (PTA),^13^ *International Classification of Diseases *(*ICD*) and self-reported hearing loss,^21^ and speech recognition in noise.^15^ Points represent genetic correlation estimates; horizontal bars represent ±1 SEs. Dashed vertical lines indicate genetic correlation of ±0.3. *P* values are from linkage disequilibrium score regression *z* tests. A Bonferroni adjustment *(P* < .0125) was used to assess significance.

Higher polygenic scores derived from the PTA-based GWAS were significantly associated with self-reported deafness (odds ratio, 1.09; 95% CI, 1.07-1.12; β = 0.087; SE=0.012*; P* = 2.18 × 10^−13^; full model Nagelkerke Pseudo-*R*^2^ = 0.15; incremental Nagelkerke pseudo-*R*^2^ = 1.04 × 10^−3^; N = 109 096) in the All of Us sample (Figure 3). Comparatively, higher polygenic scores derived from the *ICD*-based GWAS were not associated with self-reported deafness (odds ratio, 1.00; 95% CI, 0.97-1.02; β = 1.71 × 10^−3^; SE = 1.15 × 10^−2^;* P* = .88; Nagelkerke Pseudo-*R*^2^ = 0.15; incremental Nagelkerke pseudo-*R*^2^ = 1.00 × 10^−6^; N = 109 096) (Figure 3).

**Figure 3.**
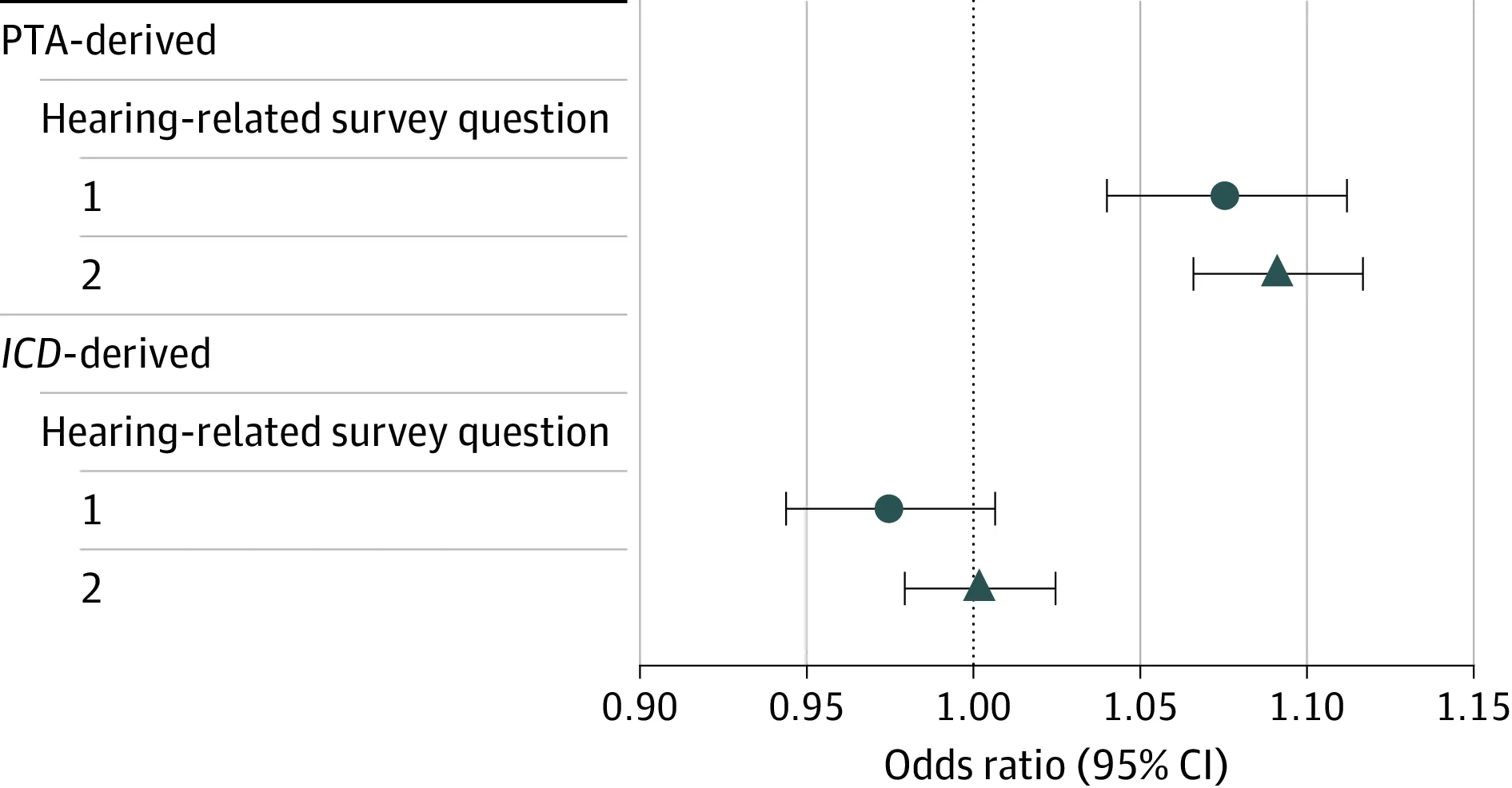
Dot Plot of Polygenic Scoring Validation in All of Us Dot plot of odds ratios from logistic regression models examining the association between polygenic risk scores and hearing loss, stratified by derivation method (pure-tone average [PTA]–derived and *International Classification of Diseases *[*ICD*]–derived) and the hearing-related survey question in the All of Us cohort, with 109 096 individuals. The dotted vertical line indicates an odds ratio of 1 (null effect).

## Discussion

The present study identified 3 genome-wide significant loci when SNHL was defined using an audiometric-based quantitative phenotype (ie, PTA), whereas no loci reached genome-wide significance when SNHL was defined using diagnostic code–based classification (*ICD*). These findings support our primary hypothesis, providing evidence for improved genetic discovery driven by the use of precision phenotypes, as contrasted by *ICD*-based binary traits. Notably, this contrast was observed despite substantially different sample sizes, with the PTA-based GWAS including 16 057 individuals and the *ICD*-based GWAS including 61 468 individuals (Table 1). To our knowledge, this study represents the largest GWAS to date using PTA as a quantitative (continuous) hearing phenotype.^13^ This difference between phenotype definitions was also reflected in SNV-based heritability estimates, with the PTA-based phenotype showing substantially higher heritability (11.78%) than the *ICD*-based phenotype (2.90%). Although the PTA-based analysis was limited by a smaller sample size, its heritability estimate falls within previously reported ranges for SNHL (approximately 2%-15%).^13^ The increased heritability of the PTA-based analysis may also be reflected by the significant, yet weak, correlation observed between *ICD* codes and PTA in the cohort shared among samples in post hoc analysis (7479 participants; point-biserial *r* = 0.106), indicating that diagnostic coding captures only a small fraction of hearing-threshold variance. Replication of quantitative precision phenotyping in larger cohorts may further refine and potentially increase the accuracy of these heritability estimates, allowing for better and more clinically meaningful genetic risk stratification for SNHL.

In addition to having higher heritability, the PTA-based loci that reached genome-wide significance mapped to genes that are biologically relevant for hearing. Specifically, the genes identified through functional annotation have all previously been found to be associated with hearing and are all integral for sensory and nervous system function (Table 2).^15^ In particular, *EYA4* acts in a monogenic cause of autosomal dominant hearing loss (DFNA10).^45^ This suggests that different variants in *EYA4* exert differential effects on hearing, with some sufficient to cause hearing loss independently and others having less severe impacts unless working in concert with other genes. Additionally, when looking at loci that reached suggestive significance *(P* < 1 × 10^−5^), functional annotation revealed 41 genomic risk loci from the PTA-based GWAS and only 21 loci from the *ICD*-based GWAS. This suggests that precision phenotyping may improve the ability to detect additional signals that may be biologically relevant. These loci represent candidates for future follow-up and replication in larger cohorts with detailed audiometric data.

The PTA-based GWAS not only replicated loci mapping to genes already associated with hearing loss in prior studies but also demonstrated strong correlation with previously reported GWASs of hearing related traits. Specifically, the PTA-based GWAS had significant genetic overlap with *ICD* and self-reported hearing loss,^21^ hearing aid use,^15^ and PTA.^13^ There was no genetic overlap, however, with speech-in-noise testing.^15^ Prior literature has shown mixed results regarding a genetic correlation between hearing loss and speech recognition in noise.^15^ Clinically, however, these are thought to represent 2 independent constructs, as difficulty in understanding speech in noise can occur independent of hearing loss, especially at the 3 frequencies captured by the PTA used.^47^ Accordingly, the observed minimal genetic overlap between hearing thresholds and speech-in-noise performance is not unexpected.

Genetic factors influencing hearing sensitivity identified through PTA-based precision phenotyping were also associated with self-reported hearing loss in an independent large cohort. Specifically, we found that PRSs from the PTA-based GWAS estimated significantly higher odds (odds ratio, 1.09; 95% CI, 1.07-1.12) of reported having hearing loss vs reporting not having hearing loss in the All of Us cohort. This contrasts with the lack of association observed between self-reported hearing loss and PRS models built exclusively on diagnostic code–based (ie, *ICD*-based) GWAS results. Thus far, PRSs used to identify variability in phenotypic hearing outcomes have been derived from large-scale GWAS meta-analyses, integrating diagnostic codes and self-reported questionnaire data capturing hearing outcomes.^18^ While our models do not generate any larger risk estimation than these previous models, the disparity between the 2 risk models generated in the present study does suggest that precision phenotyping for SNHL improves overall risk estimation. Additionally, the polygenic risk score’s association with self-reported hearing loss (odds ratio, 1.09; 95% CI, 1.07-1.12) falls in the weak credibility range (odds ratio range, 1.0-3.9) per Grimes and Schulz criteria,^49^ indicating that chance, bias, and residual confounding cannot be reliably excluded and that clinical utility for individual-level risk prediction remains unproven. Therefore, future replication of this work at comparably larger scales or in additional test datasets may generate more powerful risk models or further validate the present model.

### Strengths and Limitations

Our study leverages several important strengths, including large-scale whole-genome sequencing data linked with audiometric phenotyping and validation across a secondary cohort. These features highlight the potential of precision phenotyping to enhance SNHL prediction, although several limitations remain. First, our GWAS sample for both *ICD*-based and PTA-based phenotyping was exclusively composed of individuals of primarily European genetic ancestry, and previous studies already demonstrated poor transferability of PRS derived from a given ancestry predicting hearing outcomes in a population with a different genetic ancestry.^19^ Future studies should aim to investigate whether similar genetic associations can be found in other populations, often underrepresented in the context of clinical hearing research.^51^ Additionally, although the BioVU sample with available audiometric data was larger than those in prior comparable studies,^13^ the present findings underscore the need for closer integration of precision phenotypes, including individual measures of hearing sensitivity (eg, PTA), with genome-wide variation. Such efforts will better delineate the genetic architecture of hearing loss in the general population. Furthermore, the present study characterized hearing using a single aggregate measure (3-frequency PTA) derived from the better hearing ear. However, hearing loss varies substantially across frequency and laterality, and this variation manifests in diverse audiometric configurations that an aggregate measure may not capture. This heterogeneity is likely influenced by partially distinct genetic factors. Future research should develop more granular phenotypes of hearing sensitivity, such as individual frequency thresholds or full-audiogram based configurations that account for frequency-specific variation and the differential contributions of genetic determinants to clinically relevant hearing outcomes. Additionally, age was defined differently across cohorts based on data availability and study aims, which may introduce bias. Although defining age at first diagnosis in the diagnostic code–based cohort could plausibly skew this group toward younger age and milder disease given the progressive nature of SNHL, a diagnostic code alone does not indicate severity at diagnosis, as patients may present at varying disease stages for a range of clinical reasons. Moreover, mean (SD) age in the diagnostic code–based cohort (63.89 [21.29] years) was older than in the PTA-based cohort (55.52 [21.77] years), suggesting this approach did not systematically bias the cohort toward younger age. Additionally, we relied on a single self-reported variable for case and control definitions in All of Us, which only identified individuals reporting deafness or severe hearing loss. Although the lack of hearing data in All of Us is well recognized,^53^ this survey question likely failed to capture individuals with milder degrees of hearing loss, potentially limiting the robustness and generalizability of the PRS in subsequent populations.

## Conclusions

The results of this genetic association study suggest that precision phenotyping using audiometric data improves detection of genetic variants underlying SNHL compared with traditional diagnostic code–based approaches. The higher heritability, the greater number of genome-wide significant loci, and stronger predictive performance of PTA-derived PRS suggest that leveraging quantitative hearing measures can more accurately capture the genetic architecture of hearing sensitivity. This approach enables more robust risk stratification and lays the foundation for precision medicine–based strategies in hearing health.

## Notes

Supplement 1. **eFigure 1.** STROBE participant-flow diagrams **eFigure 2.** Distribution of Pure Tone Average (PTA)
eMethods.

Supplement 2.
Data Sharing Statement

## References

[ooi260059r1] World Report on Hearing. World Health Organization; 2021:54.

[ooi260059r2] Vas VF. *The Biopsychosocial Impact of Hearing Loss on People with Hearing Loss and Their Communication Partners*. Thesis. University of Nottingham; 2017.

[ooi260059r3] Idstad M, Engdahl B. Childhood sensorineural hearing loss and educational attainment in adulthood: results from the HUNT Study. Ear Hear. 2019;40(6):1359-1367. doi:10.1097/AUD.0000000000000716 30946138

[ooi260059r4] Emmett SD, Francis HW. The socioeconomic impact of hearing loss in US adults. Otol Neurotol. 2015;36(3):545-550. doi:10.1097/MAO.0000000000000562 25158616 PMC4466103

[5] Jung KH, Won JH, Drennan WR, . Psychoacoustic performance and music and speech perception in prelingually deafened children with cochlear implants. Audiol Neurootol. 2012;17(3):189-197. doi:10.1159/000336407 22398954 PMC3375765

[ooi260059r6] Chern A, Golub JS. Age-related hearing loss and dementia. Alzheimer Dis Assoc Disord. 2019;33(3):285-290. doi:10.1097/WAD.0000000000000325 31335455 PMC7749722

[7] Ford AH, Hankey GJ, Yeap BB, Golledge J, Flicker L, Almeida OP. Hearing loss and the risk of dementia in later life. Maturitas. 2018;112:1-11. doi:10.1016/j.maturitas.2018.03.004 29704910

[8] Kitterick PT, Edwards BE. Hearing health and dementia. Lancet Public Health. 2023;8(10):e752. doi:10.1016/S2468-2667(23)00190-1 37777282

[9] Wei J, Hu Y, Zhang L, . Hearing impairment, mild cognitive impairment, and dementia: a meta-analysis of cohort studies. Dement Geriatr Cogn Dis Extra. 2017;7(3):440-452. doi:10.1159/000485178 29430246 PMC5806170

[ooi260059r10] Fransen E, Bonneux S, Corneveaux JJ, . Genome-wide association analysis demonstrates the highly polygenic character of age-related hearing impairment. Eur J Hum Genet. 2015;23(1):110-115. doi:10.1038/ejhg.2014.56 24939585 PMC4266741

[11] Hoffmann TJ, Keats BJ, Yoshikawa N, Schaefer C, Risch N, Lustig LR. A large genome-wide association study of age-related hearing impairment using electronic health records. PLoS Genet. 2016;12(10):e1006371. doi:10.1371/journal.pgen.1006371 27764096 PMC5072625

[ooi260059r12] Ivarsdottir EV, Holm H, Benonisdottir S, . The genetic architecture of age-related hearing impairment revealed by genome-wide association analysis. Commun Biol. 2021;4(1):706. doi:10.1038/s42003-021-02224-9 34108613 PMC8190123

[ooi260059r13] Nagtegaal AP, Broer L, Zilhao NR, . Genome-wide association meta-analysis identifies five novel loci for age-related hearing impairment. Sci Rep. 2019;9(1):15192. doi:10.1038/s41598-019-51630-x 31645637 PMC6811684

[14] Praveen K, Dobbyn L, Gurski L, ; GHS-REGN DiscovEHR collaboration; Regeneron Genetics Center; Decibel-REGN collaboration. Population-scale analysis of common and rare genetic variation associated with hearing loss in adults. Commun Biol. 2022;5(1):540. doi:10.1038/s42003-022-03408-7 35661827 PMC9166757

[ooi260059r15] Liu W, Johansson Å, Rask-Andersen H, Rask-Andersen M. A combined genome-wide association and molecular study of age-related hearing loss in *H. sapiens*. BMC Med. 2021;19(1):302. doi:10.1186/s12916-021-02169-0 34847940 PMC8638543

[16] Van Laer L, Huyghe JR, Hannula S, . A genome-wide association study for age-related hearing impairment in the Saami. Eur J Hum Genet. 2010;18(6):685-693. doi:10.1038/ejhg.2009.234 20068591 PMC2987344

[17] Wells HRR, Freidin MB, Zainul Abidin FN, . GWAS identifies 44 independent associated genomic loci for self-reported adult hearing difficulty in UK Biobank. Am J Hum Genet. 2019;105(4):788-802. doi:10.1016/j.ajhg.2019.09.008 31564434 PMC6817556

[ooi260059r18] Schmitz J, Abbondanza F, Paracchini S. Genome-wide association study and polygenic risk score analysis for hearing measures in children. Am J Med Genet B Neuropsychiatr Genet. 2021;186(5):318-328. doi:10.1002/ajmg.b.32873 34476894

[ooi260059r19] Cherny SS, Livshits G, Wells HRR, . Self-reported hearing loss questions provide a good measure for genetic studies: a polygenic risk score analysis from UK Biobank. Eur J Hum Genet. 2020;28(8):1056-1065. doi:10.1038/s41431-020-0603-2 32203203 PMC7382483

[ooi260059r20] Choi JE, Moon IJ, Baek SY, Kim SW, Cho YS. Discrepancies between self-reported hearing difficulty and hearing loss diagnosed by audiometry: prevalence and associated factors in a national survey. BMJ Open. 2019;9(4):e022440. doi:10.1136/bmjopen-2018-022440 31048419 PMC6501946

[ooi260059r21] Trpchevska N, Freidin MB, Broer L, ; Estonian Biobank Research Team. Genome-wide association meta-analysis identifies 48 risk variants and highlights the role of the stria vascularis in hearing loss. Am J Hum Genet. 2022;109(6):1077-1091. doi:10.1016/j.ajhg.2022.04.010 35580588 PMC9247887

[22] Nelson SJ, Yin Y, Trujillo Rivera EA, . Are *ICD* codes reliable for observational studies? assessing coding consistency for data quality. Digit Health. 2024;10:20552076241297056. doi:10.1177/20552076241297056 39493629 PMC11528819

[23] Santin A, Pianigiani G, Gialluisi A, . Normal hearing function genetics: have you heard all about it? an integrated approach of genome-wide association studies and transcriptome-wide association studies in three Italian cohorts. Front Genet. 2025;16:1522338. doi:10.3389/fgene.2025.1522338 40458558 PMC12127661

[24] Vuckovic D, Dawson S, Scheffer DI, . Genome-wide association analysis on normal hearing function identifies *PCDH20* and *SLC28A3* as candidates for hearing function and loss. Hum Mol Genet. 2015;24(19):5655-5664. doi:10.1093/hmg/ddv279 26188009 PMC4572074

[25] Ahmed S, Vaden KI Jr, Lewis MA, . Large-scale audiometric phenotyping identifies distinct genes and pathways involved in hearing loss subtypes. Commun Biol. 2025;8(1):1515. doi:10.1038/s42003-025-08778-2 41174146 PMC12579245

[ooi260059r26] Denny JC, Rutter JL, Goldstein DB, ; All of Us Research Program Investigators. The “All of Us” Research Program. N Engl J Med. 2019;381(7):668-676. doi:10.1056/NEJMsr1809937 31412182 PMC8291101

[ooi260059r27] Wei-Qi W, Denny J. Hearing Loss. PheKB. Accessed August 28, 2024. https://phekb.org/phenotype/656

[ooi260059r28] Carhart R. Observations on relations between thresholds for pure tones and for speech. J Speech Hear Disord. 1971;36(4):476-483. doi:10.1044/jshd.3604.476 5125799

[29] Ristovska L, Jachova Z, Kovacevic J, Radovanovic V, Hasanbegovic H. Correlation between pure tone thresholds and speech thresholds. Hum Res Rehabil. 2021;11:120-125. doi:10.21554/hrr.092108

[ooi260059r30] Wilson RH. Some observations on the nature of the audiometric 4000 Hz notch: data from 3430 veterans. J Am Acad Audiol. 2011;22(1):23-33. doi:10.3766/jaaa.22.1.4 21419067

[31] Coles RR, Lutman ME, Buffin JT. Guidelines on the diagnosis of noise-induced hearing loss for medicolegal purposes. Clin Otolaryngol Allied Sci. 2000;25(4):264-273. doi:10.1046/j.1365-2273.2000.00368.x 10971532

[32] Boger ME, Barbosa-Branco A, Ottoni ÁC. The noise spectrum influence on noise-induced hearing loss prevalence in workers. Braz J Otorhinolaryngol. 2009;75(3):328-334. doi:10.1016/S1808-8694(15)30646-7 19649479 PMC9445875

[ooi260059r33] Roden DM, Pulley JM, Basford MA, . Development of a large-scale de-identified DNA biobank to enable personalized medicine. Clin Pharmacol Ther. 2008;84(3):362-369. doi:10.1038/clpt.2008.89 18500243 PMC3763939

[ooi260059r34] Avery C, Babanejad M, Baker J, . Genome sequencing of 35,024 predominantly African ancestry persons addresses gaps in genomics and healthcare. medRxiv. Preprint posted online November 2, 2025. doi:10.1101/2025.10.30.25338549

[ooi260059r35] Mbatchou J, Barnard L, Backman J, . Computationally efficient whole-genome regression for quantitative and binary traits. Nat Genet. 2021;53(7):1097-1103. doi:10.1038/s41588-021-00870-7 34017140

[ooi260059r36] Watanabe K, Taskesen E, van Bochoven A, Posthuma D. Functional mapping and annotation of genetic associations with FUMA. Nat Commun. 2017;8(1):1826. doi:10.1038/s41467-017-01261-5 29184056 PMC5705698

[ooi260059r37] Bulik-Sullivan BK, Loh PR, Finucane HK, ; Schizophrenia Working Group of the Psychiatric Genomics Consortium. LD Score regression distinguishes confounding from polygenicity in genome-wide association studies. Nat Genet. 2015;47(3):291-295. doi:10.1038/ng.3211 25642630 PMC4495769

[38] Bulik-Sullivan B, Finucane HK, Anttila V, ; ReproGen Consortium; Psychiatric Genomics Consortium; Genetic Consortium for Anorexia Nervosa of the Wellcome Trust Case Control Consortium 3. An atlas of genetic correlations across human diseases and traits. Nat Genet. 2015;47(11):1236-1241. doi:10.1038/ng.3406 26414676 PMC4797329

[ooi260059r39] Purcell S, Neale B, Todd-Brown K, . PLINK: a tool set for whole-genome association and population-based linkage analyses. Am J Hum Genet. 2007;81(3):559-575. doi:10.1086/519795 17701901 PMC1950838

[ooi260059r40] Chang CC, Chow CC, Tellier LC, Vattikuti S, Purcell SM, Lee JJ. Second-generation PLINK: rising to the challenge of larger and richer datasets. Gigascience. 2015;4(1):7. doi:10.1186/s13742-015-0047-8 25722852 PMC4342193

[ooi260059r41] Manichaikul A, Mychaleckyj JC, Rich SS, Daly K, Sale M, Chen WM. Robust relationship inference in genome-wide association studies. Bioinformatics. 2010;26(22):2867-2873. doi:10.1093/bioinformatics/btq559 20926424 PMC3025716

[42] Cornejo-Sanchez DM, Bharadwaj T, Dong R, . Mendelian non-syndromic and syndromic hearing loss genes contribute to presbycusis. Eur J Hum Genet. 2025;33(6):758-767. doi:10.1038/s41431-025-01789-x 40055553 PMC12185688

[43] De Angelis F, Zeleznik OA, Wendt FR, . Sex differences in the polygenic architecture of hearing problems in adults. Genome Med. 2023;15(1):36. doi:10.1186/s13073-023-01186-3 37165447 PMC10173489

[44] Verma A, Huffman JE, Rodriguez A, . Diversity and scale: genetic architecture of 2068 traits in the VA Million Veteran Program. Science. 2024;385(6706):eadj1182. doi:10.1126/science.adj1182 39024449 PMC12857194

[ooi260059r45] Shinagawa J, Moteki H, Nishio SY, . Prevalence and clinical features of hearing loss caused by *EYA4* variants. Sci Rep. 2020;10(1):3662. doi:10.1038/s41598-020-60259-0 32107406 PMC7046659

[46] Wolber LE, Steves CJ, Spector TD, Williams FMK. Hearing ability with age in northern European women: a new web-based approach to genetic studies. PLoS One. 2012;7(4):e35500. doi:10.1371/journal.pone.0035500 22558162 PMC3340381

[ooi260059r47] Ponsot E. Unravelling hidden hearing loss. Elife. 2024;13:e104936. doi:10.7554/eLife.104936 39636674 PMC11620737

[48] Vermiglio AJ, Soli SD, Freed DJ, Fang X. The effect of stimulus audibility on the relationship between pure-tone average and speech recognition in noise ability. J Am Acad Audiol. 2020;31(3):224-232. doi:10.3766/jaaa.19031 31274071

[ooi260059r49] Grimes DA, Schulz KF. False alarms and pseudo-epidemics: the limitations of observational epidemiology. Obstet Gynecol. 2012;120(4):920-927. doi:10.1097/AOG.0b013e31826af61a 22996110

[50] Kim MS, Naidoo D, Hazra U, . Testing the generalizability of ancestry-specific polygenic risk scores to predict prostate cancer in sub-Saharan Africa. Genome Biol. 2022;23(1):194. doi:10.1186/s13059-022-02766-z 36100952 PMC9472407

[ooi260059r51] Rouse SL, Florentine MM, Taketa E, Chan DK. Racial and ethnic disparities in genetic testing for hearing loss: a systematic review and synthesis. Hum Genet. 2022;141(3-4):485-494. doi:10.1007/s00439-021-02335-7 34494120 PMC9035011

[52] Florentine MM, Rouse SL, Stephans J, . Racial and ethnic disparities in diagnostic efficacy of comprehensive genetic testing for sensorineural hearing loss. Hum Genet. 2022;141(3-4):495-504. doi:10.1007/s00439-021-02338-4 34515852 PMC9035005

[ooi260059r53] He J, Curhan SG, Curhan GC, Polimanti R. Age-, sex-, and ancestry-specific prevalence of hearing loss in UK Biobank and All of Us Research Program. Aging Dis. 2025;17(4):2253-2259. doi:10.14336/AD.2025.0754 40681351 PMC13256383

