# Supplementary material for: Precision Phenotyping With Audiometric Data and Gene Discovery for Sensorineural Hearing Loss
Source: JAMA Otolaryngol Head Neck Surg. 2026 Oct 8:e263089. Online ahead of print. doi: 10.1001/jamaoto.2026.3089 (PMC13650696; doi:10.1001/jamaoto.2026.3089)
Supplement: Supplementary file 1 [file jamaotolaryngolheadnecksurg-e263089-s001.pdf]

Supplemental Online Content

DeFreese AJ, Rubat du Mérac T, Sheng Q, Nayak S, Jan TA. Precision phenotyping with audiometric data and gene discovery for sensorineural hearing loss. *JAMA Otolaryngol Head Neck Surg.* Published online October 8, 2026. doi:10.1001/jamaoto.2026.3089

eFigure 1. STROBE participant-flow diagrams

eFigure 2. Distribution of Pure Tone Average (PTA)

eMethods.

This supplemental material has been provided by the authors to give readers additional information about their work.

© 2026 DeFreese AJ et al. *JAMA Otolaryngology–Head & Neck Surgery*.

eFigure 1. STROBE participant-flow diagrams

eFigure 1A. STROBE participant-flow diagram for diagnostic code-based phenotyping cohort

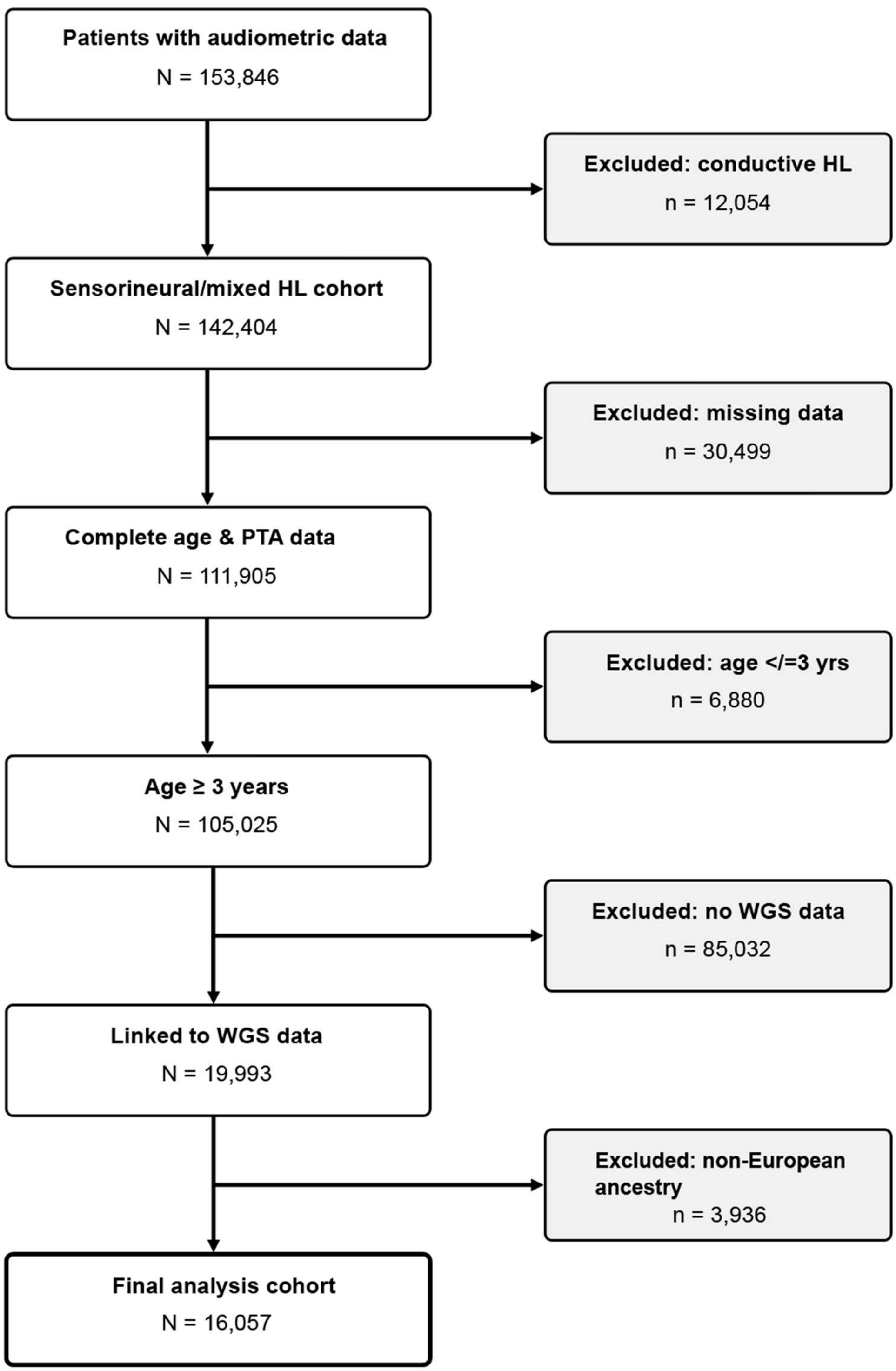

eFigure 1B. STROBE participant-flow diagram for audiogram-based precision phenotyping cohort

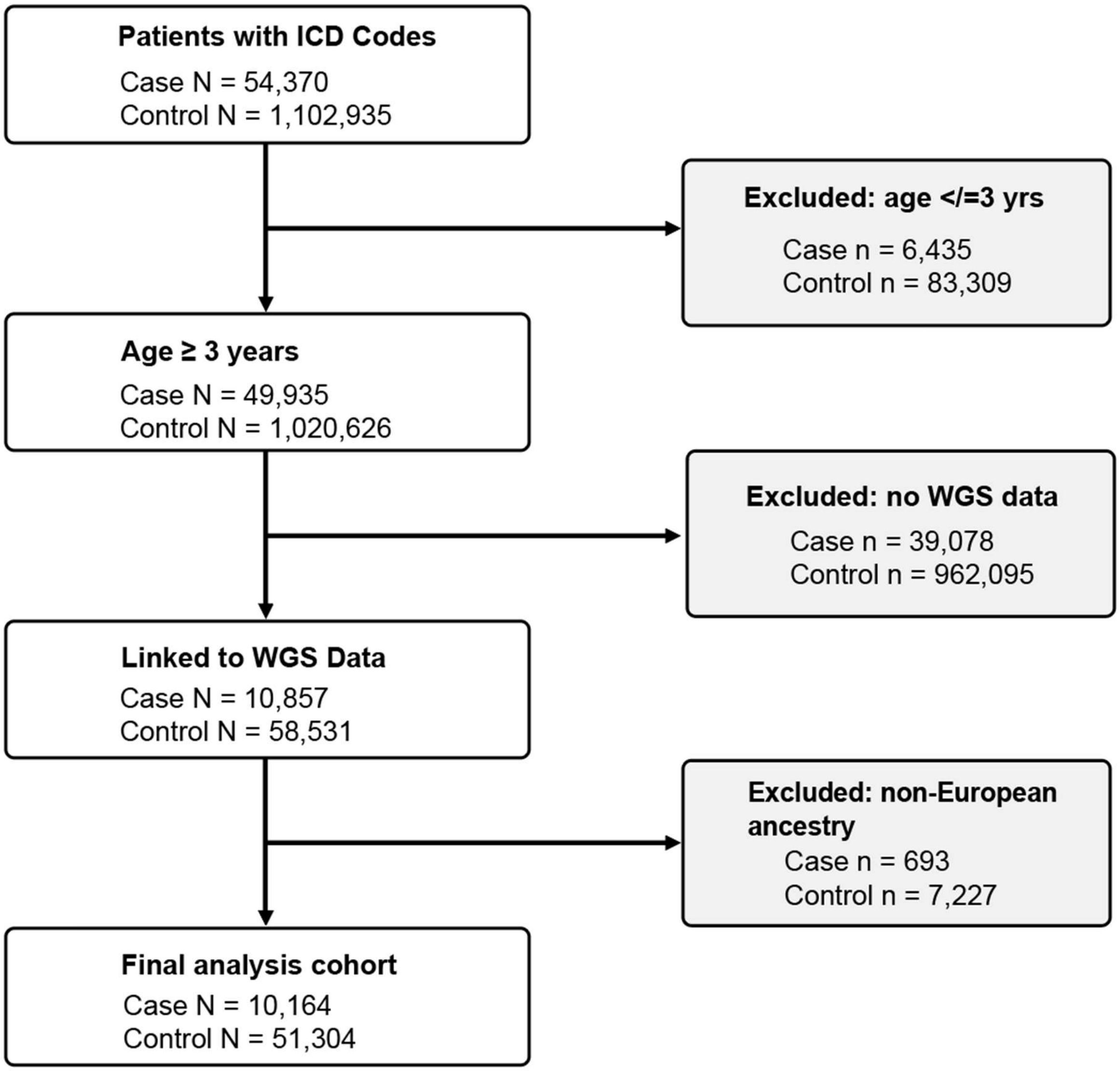

© 2026 DeFreese AJ et al. *JAMA Otolaryngology–Head & Neck Surgery*.

eFigure 2. Distribution of Pure Tone Average (PTA)

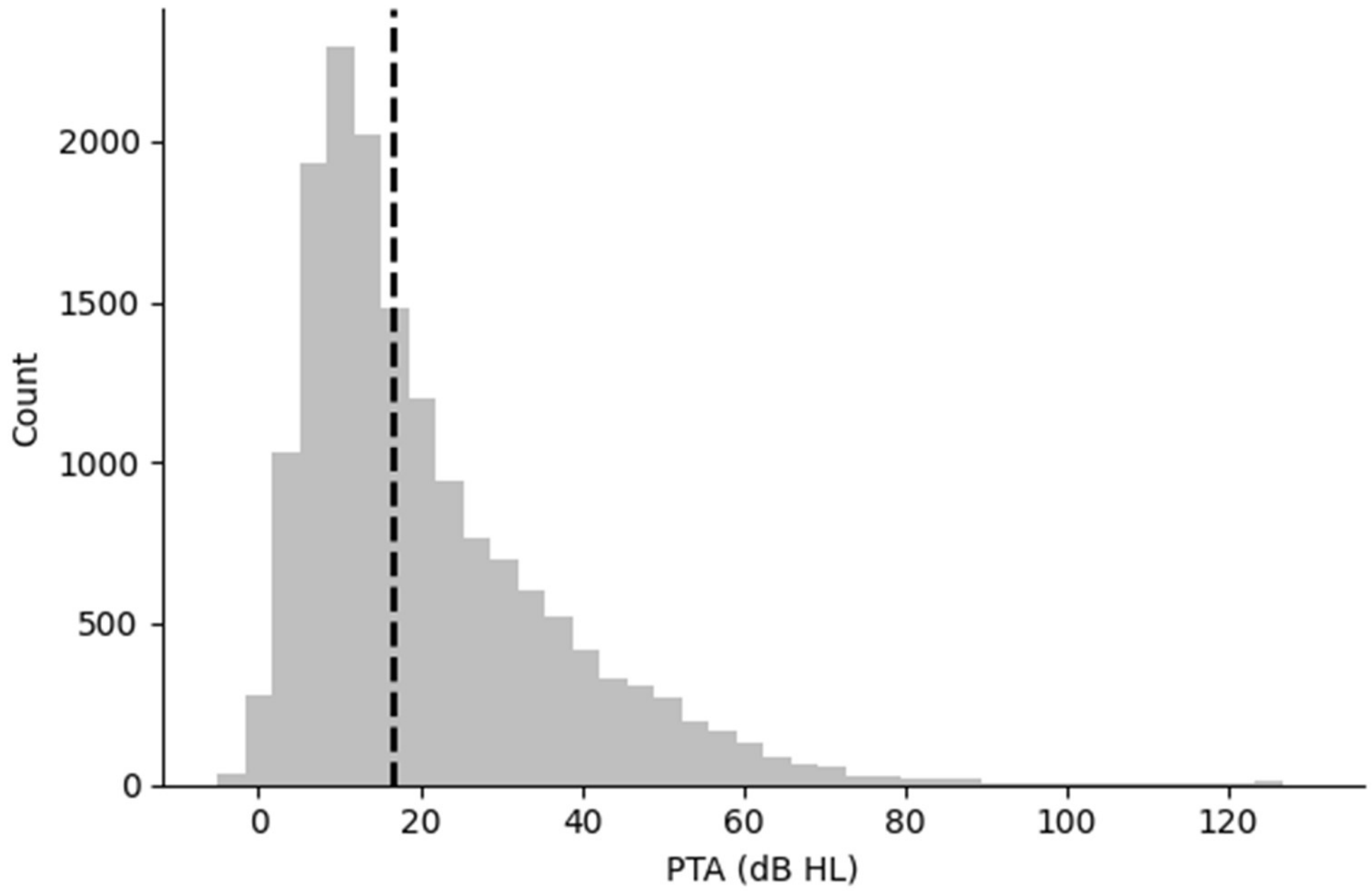

eMethods

Case-Control Definitions for Sensorineural Hearing Loss in the BioVU sample.

Cases were defined as individuals with (1) at least two diagnostic codes for sensorineural hearing loss or mixed conductive and sensorineural hearing loss recorded on separate dates, (2) at least one such diagnostic code together with a hearing device–related code, or (3) a cochlear implant procedure code. Diagnostic codes included ICD-9 codes 389.1*, 389.2*, and 389.9*, and ICD-10 codes H90.3*, H90.4*, H90.5*, H90.6*, H90.7*, and H90.8*. Hearing device-related codes included ICD-9 code V53.2 and ICD-10 codes Z46.1 and Z97.4. Cochlear implant procedures were identified using CPT code 69930. Cases were redistricted to individuals with a postlingual onset of hearing loss (e.g. age at first incidence of ICD code for sensorineural hearing loss was after the age of three.

Controls were defined as individuals who were not classified as cases, had no hearing loss–related diagnostic or procedure codes (ICD-9 codes 380–389 or V53.2; ICD-10 codes H60–H95, Z46.1, or Z97.4; CPT code 69930), and had no hearing loss–related terminology in clinical notes, including “hearing loss,” “hearing aid,” “decreased hearing,” “difficulty hearing,” “cochlear implant,” “diminished hearing,” “hard of hearing,” “loss of hearing,” “deaf,” “deafness,” or “difficulty with hearing.”

© 2026 DeFreese AJ et al. *JAMA Otolaryngology–Head & Neck Surgery*.
