# Supplementary material for: Precision Phenotyping With Audiometric Data and Gene Discovery for Sensorineural Hearing Loss
Source: JAMA Otolaryngol Head Neck Surg. 2026 Oct 8:e263089. Online ahead of print. doi: 10.1001/jamaoto.2026.3089 (PMC13650696; doi:10.1001/jamaoto.2026.3089)
Supplement: Supplementary file 2 [file jamaotolaryngolheadnecksurg-e263089-s002.pdf]

Data Sharing Statement

DeFreese. Precision Phenotyping With Audiometric Data and Gene Discovery for Sensorineural Hearing Loss. *JAMA Otolaryngol Head Neck Surg*. Published October 08, 2026. doi:10.1001/jamaoto.2026.3089

Data
Data available: No

Additional Information
Explanation for why data not available: All previously published GWAS summary statistics used in this study were all made publicly available or are accessible upon reasonable request to the corresponding authors of the original study. As part of this work, we generated two GWAS summary statistics for PTA-based and ICD-based SNGL definitions. Complete summary statistics can be accessed on the GWAS catalog website: https://www.ebi.ac.uk/gwas/. Medical information associated with the BioVU sample cannot be made publicly available due to restrictions in place to protect participants' privacy and mitigate the risk of patient re-identification. Summary stats on GWAS data will be made publicly available. Access to All of Us data and code can be requested for researchers registered on the Researcher Workbench platform (https://workbench.researchallofus.org/login). The remaining code generated as part of this study can be accessed on GitHub: https://github.com/tahajanlab/published/.
